# Boiling-Resistant Single-Chain Sweet Protein Monellin as a Safe and Effective Sugar Alternative for Metabolic and Glycemic Management in Mice

**DOI:** 10.3390/foods14213667

**Published:** 2025-10-27

**Authors:** Tingting Qi, Xiaoya Li, Lunmeng Lai, Tianjie You, Mingxue Ma, Sheng Ye, Si Liu

**Affiliations:** State Key Laboratory of Synthetic Biology, Tianjin University, Tianjin 300072, China; tingtq@tju.edu.cn (T.Q.);

**Keywords:** obesity, metabolic disorder, natural sweetener, sugar alternative, boiling-resistant monellin

## Abstract

The global rise in obesity and metabolic disorders has intensified the demand for safe and effective sugar alternatives. Monellin, a naturally sweet protein derived from *Dioscoreophyllum cumminsii*, serves as an excellent sugar alternative, but its broader application has been constrained by poor thermal stability and limited evaluation of long-term metabolic effects. In this study, we evaluated the metabolic effects of MNEI-Mut6, a boiling-resistant single-chain monellin variant, in male C57BL/6 mice fed standard chow supplemented with either 4% sucrose or an equivalent sweetness concentration of MNEI-Mut6 for 16 weeks. Compared with sucrose, MNEI-Mut6 did not promote weight gain, preserved insulin sensitivity, and maintained glucose homeostasis. In addition, MNEI-Mut6 reduced hepatic lipid accumulation and adipocyte hypertrophy without inducing hepatotoxic or nephrotoxic effects. Collectively, these findings demonstrate that MNEI-Mut6, a thermally stable and metabolically neutral sweetener, is a promising and safer alternative to sucrose and artificial sweeteners suitable for application in food processing and product formulation.

## 1. Introduction

Human preference for sweetness likely evolved from glucose’s central role as a vital energy source and regulator of blood glucose homeostasis [1]. However, excessive sugar consumption has been strongly associated with obesity, insulin resistance, and other metabolic disorders [2,3,4]. To address these health concerns, non-caloric artificial sweeteners (NAS) have been widely introduced into foods and beverages owing to their high sweetness, low caloric contribution, and reduced production costs. Nevertheless, the long-term safety and metabolic effects of NAS remain controversial [5,6,7]. The World Health Organization (WHO) recently recommended against using NAS for weight management or the prevention of non-communicable diseases, and the International Agency for Research on Cancer (IARC) classified aspartame as a possible human carcinogen [8]. These concerns highlight the urgent need for safe, natural, and effective alternatives to conventional sweeteners.

In this context, naturally sweet proteins have emerged as promising candidates [9]. They provide intense sweetness comparable to sucrose while being digested as typical dietary proteins [6]. To date, only a limited number of sweet-tasting proteins have been identified, including brazzein [10], lysozyme [11], mabinlin [12], miraculin [13], monellin [14], neoculin [15], pentadin [16], and thaumatin [17]. Among these, monellin, extracted from the African plant *Dioscoreophyllum cumminsii*, is one of the most potent, with a molar sweetness roughly 3000 times that of sucrose [18]. This small (~11 kDa) protein is composed of two non-covalently associated polypeptide chains, A (45 residues) and B (50 residues). Importantly, a recombinant monellin preparation produced by *Komagataella phaffii* P-MON-040, which expresses a modified form of the protein, received Generally Recognized As Safe (GRAS) status by the U.S. Food and Drug Administration (FDA) in December 2024 [19].

With respect to physicochemical properties, monellin exhibits poor thermal stability, losing activity irreversibly above 50 °C due to denaturation [20]. To overcome this limitation, researchers have engineered a single-chain variant known as MNEI, in which the two native chains are connected via a Gly-Phe linker, thereby improving its melting temperature to over 70 °C [21]. Building on this progress, our previous work used protein engineering to develop a more robust variant, MNEI-Mut6 (I5E/E23A/I26R/Y65I/G83E/N90D), which remains stable at 100 °C for up to an hour [22].

Concurrently, numerous toxicological studies have been conducted to provide robust evidence supporting the safety of sweet proteins [23]. Similarly, the serendipity berry sweet protein (SbSP), which shares ~95% sequence homology with monellin, was expressed in *K. phaffii* and subjected to comprehensive toxicological evaluation, demonstrating no adverse effects in vitro or in vivo [24]. In vivo investigations on the long-term consumption of recombinant brazzein and monellin in rats, showing no negative impacts on health status or gut microbiota composition [25]. Moreover, the expression of a heat-stable MNEI mutant in the milk of transgenic mice confirmed not only its functional integrity but also provided an alternative production system for large-scale application [26]. Overall, several preclinical and clinical studies have begun to explore the physiological impacts of sweet proteins in mammals and humans. These findings strongly support the safety profile of monellin and its engineered variants, yet they also highlight the need for deeper evaluation of their metabolic effects, especially regarding glucose homeostasis and insulin sensitivity.

Although the thermal stability and safety of monellin and its engineered variants are well established, their long-term metabolic effects, particularly on glucose regulation and insulin sensitivity, remain largely unexplored. Addressing this gap, the present study investigates the impact of the boiling-resistant monellin variant, MNEI-Mut6, on metabolic and glycemic outcomes in C57BL/6 male mice over a 16-week intervention. These findings will provide critical insight into the suitability of sweet proteins as functional sugar alternatives for managing obesity and metabolic disorders.

## 2. Method

### 2.1. Plasmid Construction and Pichia pastoris Transformation

The full-length wild-type *MNEI* gene (GenBank: AFF58925.1) and the MNEI-Mut6 variant, containing six amino acid substitutions (I5E, E23A, I26R, Y65I, G83E, and N90D), were synthesized with codon optimization. The following site-directed mutations were introduced: I5 (ATC → GAG), E23 (GAA → GCA), I26 (ATT → CGC), Y65 (TAC → ATA), G83 (GGT → GAA), and N90 (AAC → GAC). The recombinant expression cassettes were inserted into the pHBM905ABDM plasmid, digested with *EcoRI* and *BamHI* (Takara Bio, Kyoto, Japan).

For yeast transformation, the recombinant plasmid carrying two expression cassettes was linearized with *SalI* (Takara Bio, Kyoto, Japan) and introduced into *P. pastoris* GS115 (ZhuangMeng Biotech, Shanghai, China) by electroporation using a Bio-Rad Micropulser (10,000 V/cm, 10 µF, 600 Ω) with a 2-mm electroporation cuvette (Bio-Rad, Hercules, CA, USA) according to the manufacturer’s protocol. Transformants were selected on MD plates containing 2% glucose and 1.34% Yeast Nitrogen Base without histidine (Sangon Biotech, Shanghai, China).

### 2.2. Expression and Purification of the Protein

#### 2.2.1. Expression in *P. pastoris*

The recombinant *P. pastoris* GS115 strain was cultured in BMMY medium supplemented with 1% methanol (Sigma-Aldrich, St. Louis, MO, USA) added every 24 h for 5 days.

#### 2.2.2. Protein Purification

The culture supernatant was separated by centrifugation at 2680× *g* for 20 min using a refrigerated centrifuge (Eppendorf, Hamburg, Germany). The clarified supernatant was adjusted to 40% saturation by adding ammonium sulfate (304 g/L; Sangon Biotech, Shanghai, China). The precipitate was collected by centrifugation at 24,328× *g* for 20 min (Thermo Fisher Scientific, Waltham, MA, USA) and redissolved in 20 mM Tris-HCl, 150 mM NaCl (pH 7.4). The dialyzed solution was applied to a Capto™ Q ion exchange chromatography column (Cytiva, Uppsala, Sweden), and the eluted fractions were dialyzed against ultrapure water.

### 2.3. Thermal Shift Assay

Thermal denaturation spectra were recorded using an Applied Biosystems™ 7500 Fast Real-Time PCR System (Thermo Fisher Scientific, Waltham, MA, USA) [5]. Each 20 µL reaction mixture contained 2.5 µL 8 × Protein Thermal Shift™ Dye, 5 µL Protein Thermal Shift™ Buffer, and 12.5 µL of purified protein sample. The mixtures were loaded into 96-well 0.2 mL thin-wall PCR plates (Applied Biosystems™, Foster City, CA, USA). The denaturation profile was obtained by gradually increasing the temperature from 25 °C to 99.5 °C at a ramp rate of 0.05 °C/s. Data were analyzed using the Melt Curve application with ROX™ as the reporter dye.

### 2.4. Cell Cytotoxicity Assay

The cytotoxicity of the proteins was evaluated in L929 mouse fibroblast cells, HepG2 human hepatocellular carcinoma cells, and HEK293T human embryonic kidney cells using the Cell Counting Kit-8 (CCK-8; Dojindo, Kumamoto, Japan). Cells were cultured in Dulbecco’s Modified Eagle Medium (DMEM; Gibco, Grand Island, NY, USA) supplemented with 10% fetal bovine serum (FBS; Gibco, Grand Island, NY, USA) at 37 °C in 5% CO_2_. For assays, 5000–10,000 cells per well were seeded into 96-well flat-bottom plates (Corning, NY, USA) and incubated for 12–24 h to allow cell attachment. A range of protein concentrations (0.01–2 mg/mL) was tested. Cells were treated for 6 h, 24 h, and 48 h to evaluate both acute and longer-term effects. After treatment, 10 µL of CCK-8 solution was added to each well and incubated for 1 h. Absorbance was measured at 450 nm using a microplate reader (Tecan, Männedorf, Switzerland).

### 2.5. Mice and Diet

#### 2.5.1. Animals and Diet

Six- to eight-week-old male C57BL/6 mice (18–22 g) were obtained from Beijing Vital River Laboratory Animal Technology Co., Ltd. (Beijing, China). All animal procedures were conducted in accordance with the Guidelines for the Care and Use of Laboratory Animals and approved by the Scientific Ethics Committee of Tianjin University, China (Approval No. TJUE-2024-046). Mice were housed under SPF conditions (22 ± 2 °C, 50 ± 10% humidity, 12 h light/dark cycle) with free access to chow (Research Diets, New Brunswick, NJ, USA). Animals were randomly assigned into four groups:

- Control (n = 5, biological replicates; water, oral gavage, 10 mL/kg);
- 4% sucrose solution (n = 5, biological replicates; Sigma-Aldrich, USA);
- WT MNEI (n = 7, biological replicates; 0.0015% solution, equivalent sweetness to 4% sucrose);
- MNEI-Mut6 (n = 7, biological replicates; 0.0015% solution, equivalent sweetness to 4% sucrose).

The equivalence in sweetness was based on sensory evaluation studies reported previously, in which the perceived sweetness of MNEI and MNEI-Mut6 solutions was compared to sucrose solutions using a trained sensory panel of ten participants, following the two-alternative forced-choice method described by Kant and it is reported that EDmouse values (3.10 mg/kg for monellin), and assuming mice (20 g) consume ~4 mL/day of 15 µg/mL solution, the daily intake approximates 3 mg/kg, consistent with published estimates [23]. During the 16-week experiment, all solutions were prepared freshly with sterile distilled water, mice had free access to water in the dark cycle and were gavaged during the light cycle, and body weight was recorded weekly.

#### 2.5.2. Sample Collection

At the end of the study, mice were anesthetized with isoflurane, and blood was collected from the retro-orbital plexus. Glucose (GLU) and alanine aminotransferase (ALT) levels were measured using a Mindray BS-200E biochemical analyzer (Mindray, Shenzhen, China). After euthanasia by cervical dislocation, liver, kidney, white adipose tissue (WAT), and brown adipose tissue (BAT) were excised, weighed and preserved for histological analysis.

### 2.6. Glucose Tolerance Test and Serum Insulin Analysis

Mice were fasted for 6 h with free access to water. Fasting blood glucose was measured using an Accu-Chek Performa glucometer (Roche Diagnostics GmbH, Mannheim, Germany). For OGTT (Oral Glucose Tolerance Test), mice were gavaged with 20% glucose solution (2 g/kg body weight, Sigma-Aldrich). Tail vein blood was collected at 0, 30, 60, 90, and 120 min post-gavage. The glucose tolerance curve and AUC (Area Under the Curve) were calculated. Serum insulin levels were quantified using a Mouse Insulin ELISA Kit (JONLNBIO, Shanghai, China) per the manufacturer’s protocol. The homeostatic model assessment of insulin resistance (HOMA-IR) was calculated as [27]:

HOMA-IR = [fasting glucose (mmol/L) × fasting insulin (µU/L)]/22.5

### 2.7. Histology

Liver, kidney, WAT, and BAT were fixed in 10% neutral-buffered formalin (Sigma-Aldrich, St. Louis, MO, USA) for 24 h, embedded in paraffin, and sectioned at 5 µm using a rotary microtome (Leica Microsystems, Wetzlar, Germany). Sections were stained with hematoxylin and eosin (H&E; Servicebio, Wuhan, China). For lipid deposition, liver cryosections (8 µm) were prepared in OCT compound (Sakura Finetek, Torrance, CA, USA), frozen at −80 °C, and stained with Oil Red O (Sigma-Aldrich). Images were captured with a DP26 microscope (Olympus, Tokyo, Japan). Epididymal adipocyte diameter was analyzed with ImageJ (version 1.54 g, NIH, USA) using calibrated scale bars. At least 100 adipocytes per mouse were measured.

### 2.8. Statistics

All analyses were conducted using GraphPad Prism v9.5.1 (GraphPad Software, La Jolla, CA, USA). Data are expressed as mean ± SEM. Normality was tested using the Shapiro–Wilk test. One-way ANOVA was used for multiple group comparisons, followed by Tukey’s HSD post hoc test to identify inter-group differences. A *p*-value < 0.05 was considered statistically significant. Significance levels were represented as: * *p* < 0.05, ** *p* < 0.01, *** *p* < 0.001, **** *p* < 0.0001; ns, not significant.

## 3. Results

### 3.1. Production and Biochemical Characterization of Mnei-Mut6

**Figure 1:**
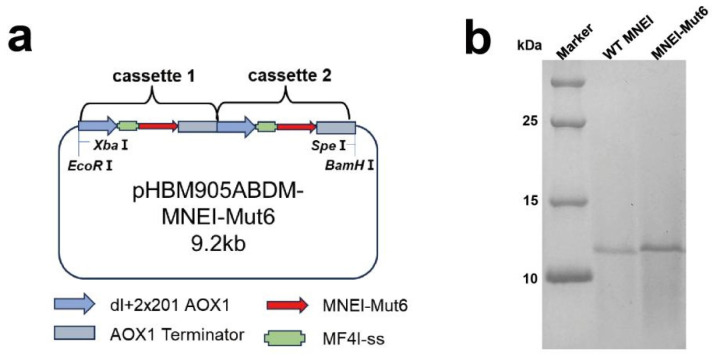
Bioproduction and biochemical properties of MNEI-Mut6. (**a**) Schematic diagram of recombinant plasmid containing MNEI-Mut6 expression cassettes. (**b**) SDS-PAGE analysis of recombinant MNEI-Mut6 and WT MNEI in *P. pastoris*.

To efficiently produce the boiling-resistant monellin variant MNEI-Mut6, a recombinant plasmid carrying two expression cassettes was constructed and transformed into *P. pastoris* GS115 (Figure 1a). After six days of induction in shake flasks, the protein was purified, and its solubility was confirmed via SDS-PAGE (Figure 1b). Compared to proteins purified from *E. coli*, thermal shift assays demonstrated that MNEI-Mut6 maintained a high melting temperature (Tm > 96 °C), consistent with its engineered thermal stability (Figure S1a,b). The cytotoxicity of MNEI-Mut6 and WT MNEI was evaluated using the CCK-8 assay in L929, HEK293T, and HepG2 cells. No significant reduction in cell viability was observed at concentrations up to 2 mg/mL after 6–48 h exposure. (Figure S2). Furthermore, proteins expressed in *P. pastoris* GS115 showed comparable sweetness thresholds to those expressed in *E. coli* when assessed by sensory evaluation (Table S1).

### 3.2. Long-Term Mnei-Mut6 Supplementation Exhibits Neutral Effects on Body Weight Compared to Sucrose

**Figure 2:**
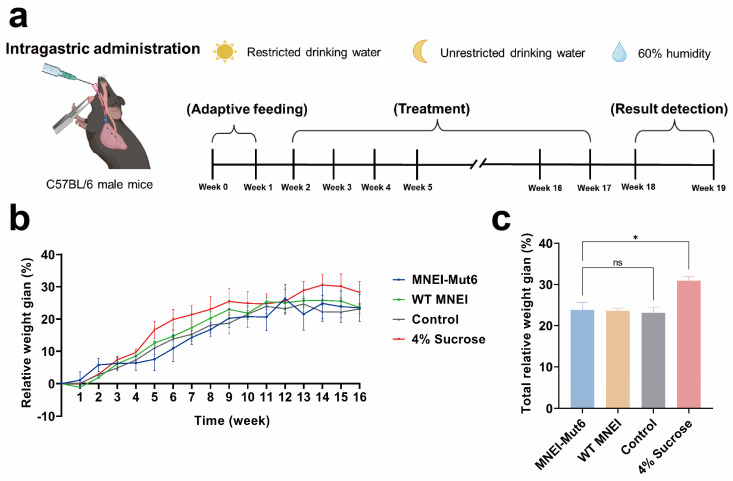
Long-term effects of MNEI-Mut6 supplementation on body weight in male C57BL/6 mice. (**a**) Schematic representation of the 16-week dietary treatment with water control (n = 5), MNEI-Mut6 (n = 7), WT MNEI (n = 7), and 4% sucrose (n = 5) supplementation in male C57BL/6 mice. (**b**) Weekly relative body weight gain during the 16-week supplementation period. (**c**) Statistical analysis of relative body weight gain in male C57BL/6 mice from Figure 2b after 16 weeks of supplementation. Data are expressed as the mean ± SEM. Analyses were performed using the one-way ANOVA, * *p* < 0.05; ns, not significant.

To evaluate the long-term effects of MNEI-Mut6, male C57BL/6 mice were given water containing WT MNEI or MNEI-Mut6 at 15 µg/mL corresponding to the sweetness of a 4% sucrose solution. Control mice received water, and the comparison group received 4% sucrose solution (Figure 2a). After 16 weeks, body weight gain was 23.12% (control group), 30.89% (sucrose group), 23.59% (WT MNEI group), and 23.81% (MNEI-Mut6 group) (Figure 2b,c). Neither WT MNEI nor MNEI-Mut6 caused significant weight gain compared to controls (*p* = 0.9771). By contrast, the sucrose group gained significantly more weight than all other groups (*p* < 0.05).

### 3.3. Long-Term Mnei-Mut6 Supplementation Maintains Glucose Homeostasis

**Figure 3:**
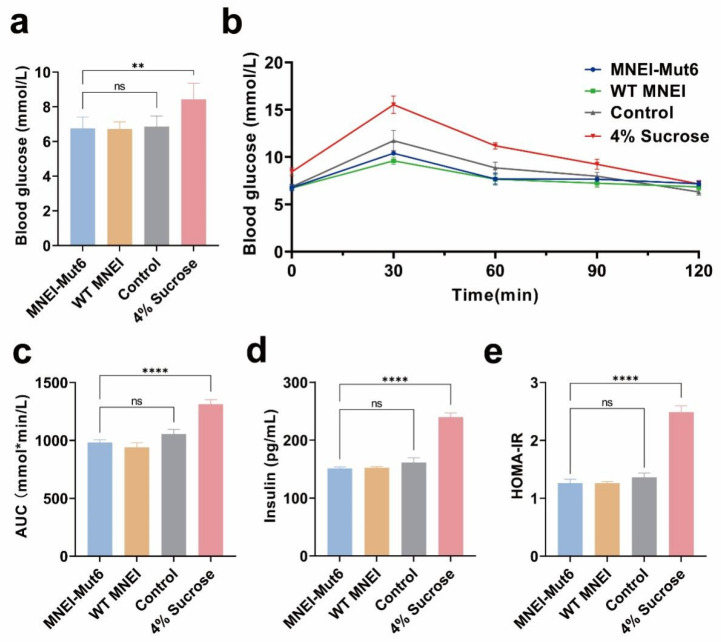
Impact of long-term MNEI-Mut6 supplementation on glucose homeostasis and insulin resistance. (**a**) Fasting blood glucose levels of mice after a 12-h fasting period. (**b**) Blood glucose levels during the glucose tolerance test (GTT). (**c**) AUC of the GTT data. (**d**) Fasting insulin levels in mice. (**e**) HOMA-IR index. Male C57BL/6 mice were supplemented with water (control, n = 5), MNEI-Mut6 (n = 7), WT MNEI (n = 7), or 4% sucrose (n = 5) for 16 weeks. Data are presented as the mean ± SEM. Statistical analyses were performed using one-way ANOVA. ** *p* < 0.01, **** *p* < 0.0001; ns, not significant.

Glucose homeostasis was assessed using OGTT after 12 h fasting. Fasting glucose levels were similar among control, WT MNEI, and MNEI-Mut6 groups (6.0–7.0 mmol/L), but were significantly higher in the sucrose group (8.44 mmol/L, *p* = 0.0032 vs. MNEI-Mut6; Figure 3a).

Following oral gavage of glucose solution (2 g/kg), blood glucose increased in all groups, but the rise was significantly greater in the sucrose group compared to MNEI-Mut6 (Figure 3b). AUC analysis confirmed impaired tolerance in sucrose-fed mice (1313.4 ± 99.9 mmol·min/L) compared with MNEI-Mut6 (981.3 ± 52.8 mmol·min/L; Figure 3c).

Fasting insulin levels were markedly elevated in the 4% sucrose group (239.64 pg/mL) compared to the control group (161.46 pg/mL), whereas the MNEI-Mut6 group showed values comparable to the control group (151.38 pg/mL) (Figure 3d).

The HOMA-IR index was significantly higher in the 4% sucrose group compared to the MNEI-Mut6 group (*p* < 0.0001), whereas the MNEI-Mut6 group showed values comparable to the control (Figure 3e).

### 3.4. Long-Term Mnei-Mut6 Supplementation Does Not Induce Liver Toxicity or Fat Accumulation

**Figure 4:**
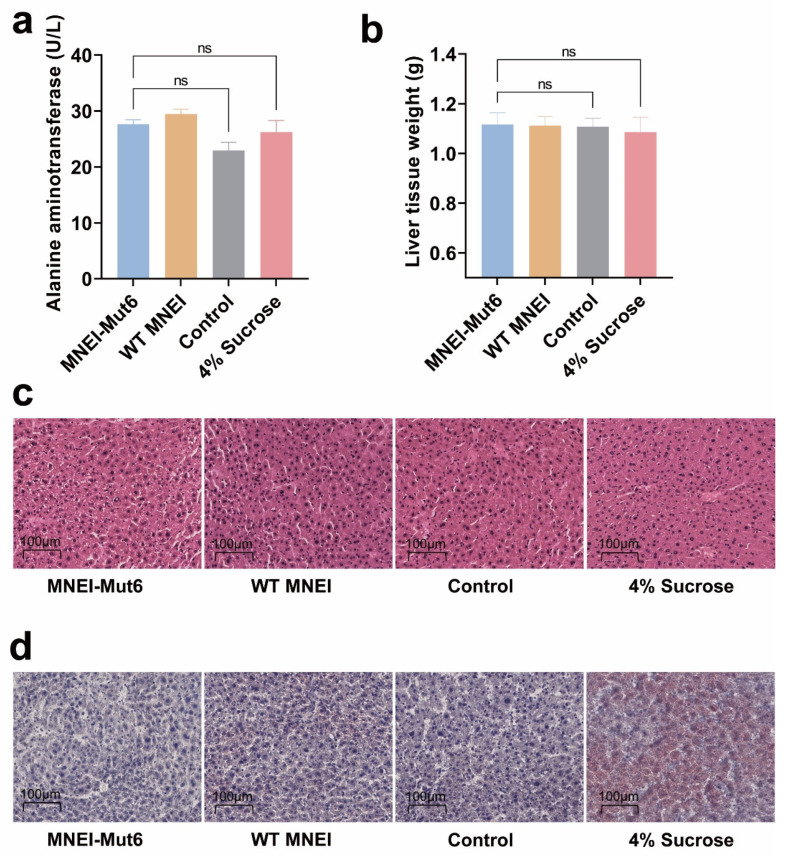
Evaluation of liver toxicity and fat accumulation after long-term supplementation. (**a**) Alanine aminotransferase (ALT) levels. (**b**) Liver wet weight. (**c**) Representative H&E-stained liver. Scale bar: 100 µm. (**d**) Representative Oil Red O-stained liver. Scale bar: 100 µm. Male C57BL/6 mice were supplemented with water (control, n = 5), MNEI-Mut6 (n = 7), WT MNEI (n = 7), or 4% sucrose (n = 5) for 16 weeks. Data are presented as the mean ± SEM. Statistical analyses were performed using one-way ANOVA. ns, not significant.

Serum ALT levels in the MNEI-Mut6 group (27.65 U/L) remained within the normal range for male C57BL/6 mice (14–38 U/L) and did not differ significantly from controls (Figure 4a). Liver weights were similar across groups (*p* = 0.9985; Figure 4b) [28].

Histological evaluation using H&E staining revealed no pathological abnormalities in the MNEI-Mut6 group (Figure 4c). Oil Red staining confirmed the absence of hepatic lipid accumulation in MNEI-Mut6-treated mice, in contrast to the pronounced lipid deposition observed in the 4% sucrose group (Figure 4d).

These data support that MNEI-Mut6 does not cause hepatotoxicity or promote hepatic steatosis under long-term supplementation.

### 3.5. Long-Term Mnei-Mut6 Supplementation Mitigates Fat Accumulation

**Figure 5:**
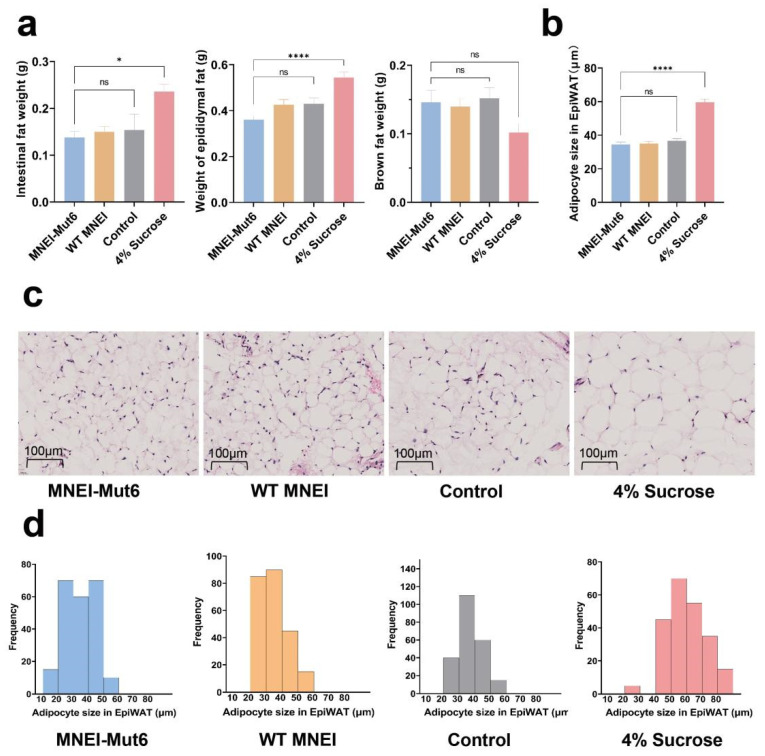
Impact of long-term MNEI-Mut6 supplementation on fat distribution and adipocyte morphology. (**a**) Weight of dissected fat depots. Mesenteric white adipose tissue (MesWAT), epididymal white adipose tissue (EpiWAT), and brown adipose tissue (BAT) weights from mice after 16 weeks of supplementation with water (control, n = 5), 4% sucrose (n = 5), WT MNEI (n = 7), or MNEI-Mut6 (n = 7). (**b**) Quantitative analysis of adipocyte size in EpiWAT. (**c**) Representative H&E-stained EpiWAT in each group. (**d**) Histogram of adipocyte size ranges in each group. Scale bar: 100 µm. Data are presented as mean ± SEM. Statistical analyses were performed using one-way ANOVA. * *p* < 0.05, **** *p* < 0.0001; ns, not significant.

To investigate adiposity, mesenteric white adipose tissue (MesWAT), epididymal white adipose tissue (EpiWAT), and brown adipose tissue (BAT) were weighed after 16 weeks. MNEI-Mut6 mice had WAT weights comparable to controls, whereas sucrose-fed mice showed significant increases in MesWAT (*p* = 0.0116) and EpiWAT (*p* < 0.0001). BAT weight was higher in MNEI-Mut6 compared with sucrose (Figure 5a).

Histological analysis of EpiWAT revealed distinct differences in adipocyte size across the groups. In the control group, 93.33% of adipocytes had diameters within the range of 20–50 µm. Similarly, the MNEI-Mut6 group exhibited adipocyte diameters predominantly between 20 and 50 µm, with 88.89% of cells falling within this range. In contrast, adipocytes in the 4% sucrose group displayed a broader size distribution (20–100 µm), with 73.33% of adipocytes exceeding 50 µm in diameter, indicating significant hypertrophy (Figure 5b–d).

### 3.6. Long-Term Mnei-Mut6 Supplementation Does Not Impair Renal Structure or Function

**Figure 6:**
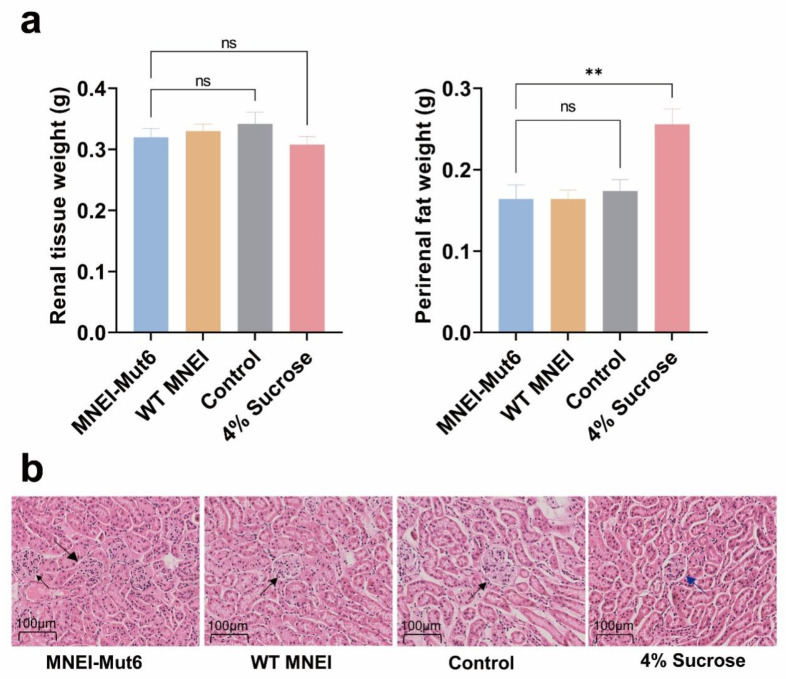
Evaluation of kidney health and perirenal fat accumulation following long-term MNEI-Mut6 supplementation. (**a**) Kidney weight and perirenal fat weight. (**b**) Representative H&E-stained kidney tissue in each group. Normal glomerulus (black arrow), crumpled glomerulus (blue arrow). Scale bar: 100 µm. Male C57BL/6 mice were supplemented with water (control, n = 5), MNEI-Mut6 (n = 7), WT MNEI (n = 7), or 4% sucrose (n = 5) for 16 weeks. Data are presented as the mean ± SEM. Statistical analyses were performed using one-way ANOVA. ** *p* < 0.01; ns, not significant.

Quantification of perirenal fat revealed no significant difference between the MNEI-Mut6 and control groups. In contrast, the 4% sucrose group exhibited a substantial increase in perirenal fat accumulation (*p* = 0.0018), suggesting that sucrose consumption promotes regional fat deposition (Figure 6a).

To assess the potential impact of MNEI-Mut6 supplementation on kidney health, we performed histological examination of kidney tissue using H&E staining and quantified perirenal fat accumulation. Kidney sections from control, WT MNEI and MNEI-Mut6 groups were analyzed at 40× magnification, revealing no significant differences in cellular morphology or the size and structure of glomeruli and tubules. However, In the 4% sucrose group, glomeruli exhibited mild irregularities in contour and reduced Bowman’s space compared with the normal, well-defined glomerular structures observed in the control, WT MNEI, and MNEI-Mut6 groups. These subtle morphological changes were not accompanied by inflammatory infiltration or tubular degeneration, suggesting no evident renal toxicity from MNEI-Mut6 supplementation (Figure 6b).

## 4. Discussion

Excessive carbohydrate intake is a major driver of obesity and diabetes, creating an urgent need for safe and effective sugar substitutes. In response, the food industry has developed a variety of sweeteners, including sugars, sugar alcohols, artificial sweeteners, and other compounds [28]. Although several low-calorie artificial sweeteners, such as aspartame, sucralose, and saccharin, have been approved by regulatory agencies, concerns about their long-term health effects remain. Recent studies indicate that some artificial sweeteners may disrupt the gut microbiome and glucose regulation, highlighting the need for safer, natural alternatives [5,29].

Sweet-tasting proteins are emerging as promising natural sweeteners because they provide intense sweetness through interaction with T1R2/T1R3 receptors without adding calories [30]. Among them, brazzein and thaumatin are particularly notable for their heat stability, solubility, and wide pH tolerance. In addition to sweetness, these proteins exhibit antimicrobial, antioxidant, and anti-inflammatory activities [29,31]. Unlike high sugar intake, which promotes obesity and metabolic dysfunction, sweet proteins appear metabolically neutral and safe, making them attractive candidates for food and beverage applications [7,32].

Monellin, derived from the African plant *Dioscoreophyllum cumminsii*, is one of the most potent natural sweeteners. However, its poor thermal stability has limited its broader use in food production. To address this limitation, thermostable variants such as MNEI-Mut6 have been engineered, but their long-term metabolic effects remain insufficiently explored [33,34,35]. In this study, we systematically evaluated the long-term metabolic safety and efficacy of MNEI-Mut6 by assessing its effects on metabolic health, liver function, fat accumulation, and kidney health in male C57BL/6 mice.

One key aspect of this study was assessing the impact of MNEI-Mut6 on glucose homeostasis. Fasting blood glucose and insulin levels in the MNEI-Mut6 group were comparable to those in the control group, and the OGTT results indicated no impairments in glucose clearance. In contrast, sucrose consumption markedly exacerbated glucose intolerance, indicating that MNEI-Mut6 supplementation does not induce insulin resistance. The preservation of glucose homeostasis further supports the potential of MNEI-Mut6 to mitigate metabolic disturbances typically associated with excessive sugar intake. Future mechanistic studies should examine whether MNEI-Mut6 modulates insulin sensitivity through pathways involving IRS-1/AKT signaling or gut hormone secretion (e.g., GLP-1) [36]. Although, inflammation-related cytokines and adipokines such as interleukin-6 (IL-6) and leptin were not measured in the present study, the absence of adipocyte hypertrophy and hepatic lipid accumulation in MNEI-Mut6-treated mice suggests reduced proinflammatory and adipokine-mediated activity. Integrating IL-6, leptin, and other inflammatory markers in future work will help clarify the anti-inflammatory and insulin-sensitizing mechanisms of MNEI-Mut6 [37,38].

Given the liver’s central role in metabolic regulation and its susceptibility to dietary toxicants, this study specifically evaluated potential hepatic adverse effects associated with MNEI-Mut6 supplementation. The results demonstrated that MNEI-Mut6 did not elevate serum ALT levels beyond the physiological range, nor did it alter liver morphology or relative liver weight. These findings indicate an absence of hepatic toxicity, consistent with previous reports on protein-based sweeteners. Furthermore, lipid staining revealed no hepatic fat accumulation in the MNEI-Mut6 group, whereas the sucrose group exhibited pronounced steatosis. Collectively, these data suggest that replacing sucrose with MNEI-Mut6 may reduce the risk of non-alcoholic fatty liver disease. In addition, serum adipokines such as leptin and adiponectin play essential roles in regulating energy balance and insulin sensitivity. Although these parameters were not assessed in the present study because of limited sample volume, future investigations integrating leptin and adiponectin measurements with gene expression profiling and histological analyses would provide a more comprehensive understanding of the molecular mechanisms underlying the metabolic effects of MNEI-Mut6.

In addition, regional fat deposition and adipocyte hypertrophy are key indicators of obesity-related metabolic dysfunction. Our data showed that mesenteric and epididymal fat weights in the MNEI-Mut6 group were similar to those in the control group, whereas the sucrose group exhibited significant fat accumulation. Interestingly, the MNEI-Mut6 group had higher BAT weight than the sucrose group, suggesting a potential role of MNEI-Mut6 in enhancing metabolic activity and promoting energy expenditure. Histological analysis of EpiWAT further confirmed that MNEI-Mut6 did not lead to adipocyte hypertrophy, unlike sucrose, which caused marked adipocyte enlargement. These findings underscore the potential of MNEI-Mut6 to prevent obesity and related metabolic disorders by limiting fat deposition and supporting healthier adipocyte morphology.

Since that proteins are metabolized and excreted through the kidneys, the potential nephrotoxic effects of MNEI-Mut6 were also investigated. Histological examination revealed no structural abnormalities or signs of inflammation in the kidneys of MNEI-Mut6-supplemented mice. Additionally, the absence of excessive perirenal fat accumulation further supports the safety of MNEI-Mut6 with respect to renal health. These results are consistent with its favorable metabolic profile, reinforcing its potential as a safe and effective sugar substitute without harmful effects on kidney health.

While the results are encouraging, several limitations merit consideration. First, the study was conducted exclusively in male C57BL/6 mice; sex-specific responses and interspecies differences warrant further investigation. Second, although a 16-week duration represents a substantial period in murine models, longer-term studies are necessary to fully establish chronic safety. Third, molecular mechanisms were not directly examined, future studies incorporating transcriptomic, proteomic, and metabolomic analyses will be essential to elucidate these pathways. Fourth, this study relies on a single dose of MNEI-Mut6 (sweetness-equivalent to 4% sucrose), which limits interpretation of dose–response relationships, threshold effects, safety margins (NOAEL/LOAEL), and optimal efficacy. Finally, translation to humans requires clinical validation, including comprehensive assessments of palatability, gastrointestinal tolerance, and metabolic outcomes.

## 5. Conclusions

In summary, this study shows that MNEI-Mut6 supplementation maintains glucose homeostasis, liver function, and kidney health while avoiding the harmful effects of sucrose on fat accumulation and adipocyte enlargement. Beyond its physiological safety, the superior heat stability and neutral metabolic profile of MNEI-Mut6 suggest strong potential for its incorporation into real food matrices. Owing to its resistance to denaturation up to 100 °C, MNEI-Mut6 could be applied in a wide range of heat-processed foods, including baked goods, dairy formulations, and ready-to-drink beverages, without loss of sweetness or function. Moreover, as a protein-based sweetener, MNEI-Mut6 can integrate compatibly with protein- or polysaccharide-rich systems, potentially improving texture and flavor synergy. Nonetheless, future work should evaluate its structural stability, sweetness retention, and sensory characteristics under various food processing and storage conditions. Such studies will be crucial for advancing the practical application of MNEI-Mut6 as a next-generation natural sweetener for the food industry.

## Data Availability

All data supporting the conclusions of this study are included within the manuscript. Additional information is available upon request from the corresponding authors.

## Supplementary Materials

The following supporting information can be downloaded at: https://www.mdpi.com/article/10.3390/foods14213667/s1, Figure S1: Thermostability analysis of the WT MNEI and MNEI-Mut6.; Figure S2. Cytotoxicity assessment of MNEI-Mut6 and WT MNEI using the CCK-8 assay. Table S1: Sweetness threshold of MNEI-Mut6 and WT MNEI.

## References

[1] McCaughey S.A. (2008). The taste of sugars. Neurosci. Biobehav. Rev..

[2] Malik V.S., Popkin B.M., Bray G.A., Despres J.P., Willett W.C., Hu F.B. (2010). Sugar-sweetened beverages and risk of metabolic syndrome and type 2 diabetes: A meta-analysis. Diabetes Care.

[3] Bray G.A., Nielsen S.J., Popkin B.M. (2004). Consumption of high-fructose corn syrup in beverages may play a role in the epidemic of obesity. Am. J. Clin. Nutr..

[4] Stanhope K.L., Bremer A.A., Medici V., Nakajima K., Ito Y., Nakano T., Chen G., Fong T.H., Lee V., Menorca R.I. (2011). Consumption of fructose and high fructose corn syrup increase postprandial triglycerides, LDL-cholesterol, and apolipoprotein-B in young men and women. J. Clin. Endocrinol. Metab..

[5] Huynh K., Partch C.L. (2015). Analysis of protein stability and ligand interactions by thermal shift assay. Curr. Protoc. Protein Sci..

[6] Kant R. (2005). Sweet proteins--potential replacement for artificial low calorie sweeteners. Nutr. J..

[7] Kim H., Kang J., Hong S., Jo S., Noh H., Kang B.H., Park S., Seo Y.J., Kong K.H., Hong S. (2020). 3M-Brazzein as a Natural Sugar Substitute Attenuates Obesity, Metabolic Disorder, and Inflammation. J. Agric. Food Chem..

[8] WHO (2023). Use of Non-Sugar Sweeteners: WHO Guideline.

[9] Xue H., Kang X., Hong K., Gao Y., Tang Y., Lin Y., Liu X., Huang W., Zhan J., You Y. (2025). Effects of artificial and natural sweeteners on host metabolic health: A double-edged sword. Food Res. Int..

[10] Ming D., Hellekant G. (1994). Brazzein, a new high-potency thermostable sweet protein from *Pentadiplandra brazzeana* B. FEBS Lett..

[11] Masuda T., Ueno Y., Kitabatake N. (2001). Sweetness and enzymatic activity of lysozyme. J. Agric. Food Chem..

[12] Liu X., Maeda S., Hu Z., Aiuchi T., Nakaya K., Kurihara Y. (1993). Purification, complete amino acid sequence and structural characterization of the heat-stable sweet protein, mabinlin II. Eur. J. Biochem..

[13] Kurihara K., Beidler L.M. (1968). Taste-modifying protein from miracle fruit. Science.

[14] Morris J.A., Cagan R.H. (1972). Purification of monellin, the sweet principle of *Dioscoreophyllum cumminsii*. Biochim. Biophys. Acta (BBA)-Gen. Subj..

[15] Shirasuka Y., Nakajima K., Asakura T., Yamashita H., Yamamoto A., Hata S., Nagata S., Abo M., Sorimachi H., Abe K. (2004). Neoculin as a new taste-modifying protein occurring in the fruit of *Curculigo latifolia*. Biosci. Biotechnol. Biochem..

[16] der Wel H.v., Larson G., Hladik A., Hladik C.M., Hellekant G., Glaser D. (1989). Isolation and characterization of pentadin, the sweet principle of Pentadiplandra brazzeana Baillon. Chem. Senses.

[17] van der Wel H., Loeve K. (1972). Isolation and characterization of thaumatin I and II, the sweet-tasting proteins from Thaumatococcus daniellii Benth. Eur. J. Biochem..

[18] Ogata C., Hatada M., Tomlinson G., Shin W.C., Kim S.H. (1987). Crystal structure of the intensely sweet protein monellin. Nature.

[19] U.S. Food and Drug Administration FDA FOIA Log—December 2024. https://www.fda.gov/media/184910/download.

[20] Kim S.-H., Kang C.-H., Kim R., Cho J.M., Lee Y.-B., Lee T.-K. (1989). Redesigning a sweet protein: Increased stability and renaturability. Protein Eng. Des. Sel..

[21] Tancredi T., Iijima H., Saviano G., Amodeo P., Temussi P.A. (1992). Structural determination of the active site of a sweet protein A1H NMR investigation of pMNEI. FEBS Lett..

[22] Liu Y., Xu J., Ma M., You T., Ye S., Liu S. (2024). Computational design towards a boiling-resistant single-chain sweet protein monellin. Food Chem..

[23] Novik T.S., Koveshnikova E.I., Kotlobay A.A., Sycheva L.P., Kurochkina K.G., Averina O.A., Belopolskaya M.V., Sergiev P.V., Dontsova O.A., Lazarev V.N. (2023). Sweet-Tasting Natural Proteins Brazzein and Monellin: Safe Sugar Substitutes for the Food Industry. Foods.

[24] Lifshitz Y., Paz S., Saban R., Zuker I., Shmuely H., Gorshkov K., Meetro J., Tafazoli S., Vo T., Amiram G. (2025). Safety Evaluation of Serendipity Berry Sweet Protein from Komagataella phaffii. J. Appl. Toxicol..

[25] Veselovsky V.A., Boldyreva D.I., Olekhnovich E.I., Klimina K.M., Babenko V.V., Zakharevich N.V., Larin A.K., Morozov M.D., Zoruk P.Y., Sergiev P.V. (2024). Effect of the consumption of brazzein and monellin, two recombinant sweet-tasting proteins, on rat gut microbiota. Front. Nutr..

[26] Lu R., Li X., Hu J., Wang Y., Jin L. (2024). Expression of a single-chain monellin (MNEI) mutant with enhanced stability in transgenic mice milk. Transgenic Res..

[27] Wallace T.M., Levy J.C., Matthews D.R. (2004). Use and abuse of HOMA modeling. Diabetes Care.

[28] Kurtz D.M., Travlos G.S. (2017). The Clinical Chemistry of Laboratory Animals.

[29] Chung J.H., Kong J.N., Choi H.E., Kong K.H. (2018). Antioxidant, anti-inflammatory, and anti-allergic activities of the sweet-tasting protein brazzein. Food Chem..

[30] Lu S., Chang S., Wang Y., Liu B. (2025). Advances in genetic engineering and molecular modification of sweet-tasting proteins. Chin. J. Biotechnol..

[31] Richter P., Sebald K., Fischer K., Schnieke A., Jlilati M., Mittermeier-Klessinger V., Somoza V. (2024). Gastric digestion of the sweet-tasting plant protein thaumatin releases bitter peptides that reduce H. pylori induced pro-inflammatory IL-17A release via the TAS2R16 bitter taste receptor. Food Chem..

[32] Hong S., Kim H., Kong K.-H., Hong S. (2024). 3M-Brazzein as a natural sugar substitute rescued obesity in maternal and offspring mice. J. Funct. Foods.

[33] Delfi M., Emendato A., Leone S., Lampitella E.A., Porcaro P., Cardinale G., Petraccone L., Picone D. (2021). A Super Stable Mutant of the Plant Protein Monellin Endowed with Enhanced Sweetness. Life.

[34] Song X., Yi Y., Liu L., He M., Deng S., Tian H., Yao W., Gao X. (2020). Design and development of a high temperature stable sweet protein base on monellin. Process Biochem..

[35] Leone S., Pica A., Merlino A., Sannino F., Temussi P.A., Picone D. (2016). Sweeter and stronger: Enhancing sweetness and stability of the single chain monellin MNEI through molecular design. Sci. Rep..

[36] Samuel Varman T., Shulman Gerald I. (2012). Mechanisms for Insulin Resistance: Common Threads and Missing Links. Cell.

[37] Park H.K., Ahima R.S. (2014). Leptin signaling. F1000prime Rep..

[38] Hotamisligil G.S. (2017). Inflammation, metaflammation and immunometabolic disorders. Nature.

