# Supplementary material for: Boiling-Resistant Single-Chain Sweet Protein Monellin as a Safe and Effective Sugar Alternative for Metabolic and Glycemic Management in Mice
Source: Foods. 2025 Oct 27;14(21):3667. doi: 10.3390/foods14213667 (PMC12609334; doi:10.3390/foods14213667)
Supplement: Supplementary file 1 [file foods-14-03667-s001.zip › foods-3932401-supplementary.pdf]

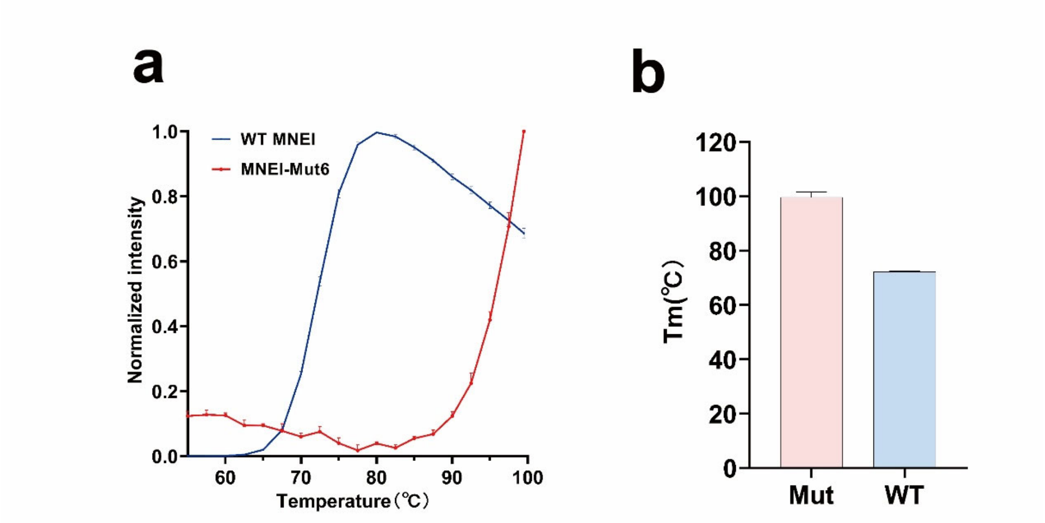

Figure S1. **Thermostability analysis of the WT MNEI and MNEI-Mut6.** (a) Thermal denaturation curves of MNEI-Mut6 and WT MNEI. (b) Bar graph illustrating T_m for WT MNEI and Mut MNEI. Data are presented as the mean ± SEM.

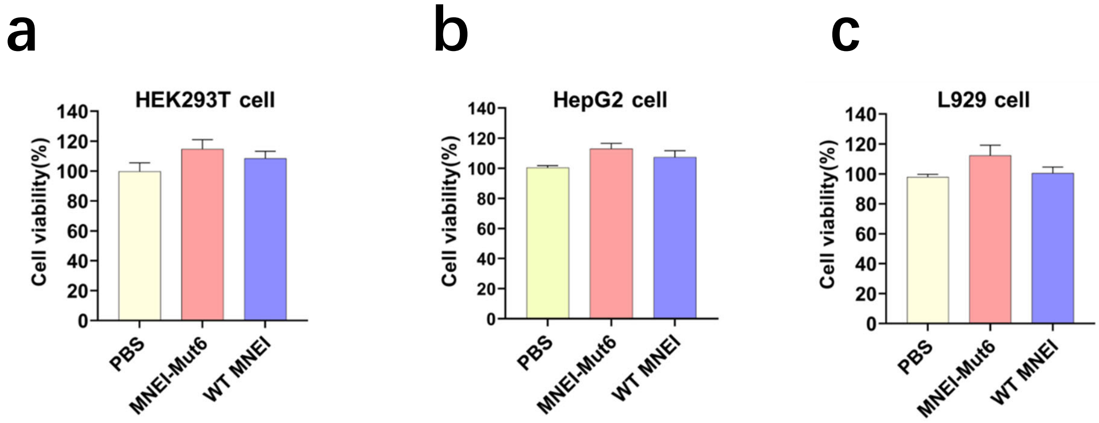

Figure S2. **Cytotoxicity assessment of MNEI-Mut6 and WT MNEI using the CCK-8 assay.** (a) in HEK293T cells with 0.5mg/mL protein for 24 h treatment; (b) in HepG2 hepatocarcinoma cells with 0.5mg/mL protein for 24 h treatment; (c) in L929 fibroblasts with 0.5mg/mL protein for 24 h treatment. Data are presented as the mean ± SEM.

Table S1 Sweetness threshold of MNEI-Mut6 and WT MNEI.

Variant (From *P. pastoris*)	Sweetness threshold (μg/mL)	Variant (From *E.coli*)	Sweetness threshold (μg/mL)
WT MNEI	12.0±3.9	WT MNEI	10.0±4.9
MNEI-Mut6	13.25±5.4	MNEI-Mut6	10.5±4.7
